# Prevalence, Severity Patterns and Risk Factors of Bronchopulmonary Dysplasia in Preterm Infants Younger than 32 Weeks of Gestation in a Tertiary Centre in Oman

**DOI:** 10.18295/squmj.3.2024.023

**Published:** 2024-05-27

**Authors:** Hilal Al Mandhari, Ashfaq Khan, Abdulrahman Al Saadi, Mazen AboAnza, Syed G.A. Rizvi, Sathiya M. Panchatcharam, Mohammed Abdulatif, Shatha Al Qassabi, Shirley Quach

**Affiliations:** 1 Child Health Department, Sultan Qaboos University Hospital, Muscat, Oman; 2 Department of Family Medicine & Public Health, Sultan Qaboos University, Muscat, Oman; 4 Family Medicine, Muscat, Oman; 3 Oman Medical Specialty Board, Muscat, Oman; 5 Department of Respiratory Therapy, SickKids Research Institute, Toronto, Canada

**Keywords:** Infant, Premature Birth, Bronchopulmonary Dysplasia, Risk Factors, Oman

## Abstract

**Objectives:**

This study aimed to determine the rate and severity patterns of bronchopulmonary dysplasia (BPD) and identify antenatal and postnatal factors associated with BPD in preterm infants <32 weeks of gestational age (GA).

**Methods:**

This retrospective observational study included preterm neonates <32 weeks of gestation admitted into the neonatal intensive care unit between January 2010 and December 2017 at Sultan Qaboos University Hospital, Muscat, Oman. A data set of antenatal and perinatal factors were collected. BPD was defined as the need for oxygen and/or respiratory support at 36 weeks post-menstrual age (PMA). Infants with and without BPD were compared in their antenatal and perinatal factors.

**Results:**

A total of 589 preterm infants <32 weeks were admitted. Among them, 505 (85.7%) survived to 36 weeks’ PMA and 90 (17.8%) had BPD. The combined BPD and mortality rate was 28.4%. Grades 1, 2 and 3 BPD constituted 77.8%, 7.8% and 14.4%, respectively. BPD was associated with lower GA, lower birth weight, need for intubation at resuscitation, lower Apgar scores, longer duration of ventilation, surfactant therapy and higher rates of neonatal morbidities. On binary logistic regression analysis, predictors of BPD were longer duration of ventilation, intraventricular haemorrhage (IVH) and necrotising enterocolitis (NEC).

**Conclusion:**

In an Omani centre, 17.8% of preterm infants (<32 weeks GA) developed BPD. Various perinatal and neonatal factors were associated with BPD. However, longer duration of ventilation, IVH grades 1 and 2 and NEC stages II and III were significant predictors. Future multicentre research is necessary to provide the overall prevalence of BPD in Oman to help optimise the resources for BPD prevention and management in preterm infants.

**Advances in Knowledge**

- *Bronchopulmonary dysplasia (BPD) in preterm infants <32 weeks gestational age on oxygen and/or respiratory support was 17.8% at 36 weeks post-menstrual age in a level III neonatal intensive care unit at a single centre in Oman.*
- *The development of BPD is associated with various perinatal and neonatal factors.*
- *Longer duration of invasive mechanical ventilation, interventricular haemorrhage and necrotising enterocolitis are the most significant predictors of BPD.*

**Application to Patient Care**

- *This study contributes to the knowledge of identifying infants at risk for BPD and enables healthcare providers to identify high-risk populations and effectively tailor interventions and implement preventative measures.*
- *The findings help empower local medical professionals to optimise overall care strategies for high-risk groups of infants at risk for BPD.*

Bronchopulmonary dysplasia (BPD) continues to be one of the major comorbidities of prematurity, a chronic lung disease affecting preterm infants exposed to prolonged oxygen and mechanical ventilation.^1^ Advancement in neonatal care over the years has led to improved survival rates of extremely and very preterm infants with BPD, described as needing oxygen and/or respiratory support at a post-menstrual age (PMA) of 36 weeks.^2^^,^^3^ The definition and classification of BPD have undergone multiple revisions.^2^^,^^4^–^7^ The most widely used definition is that of the National Institute of Child Health and Human Development (NICHD) Workshop, which was recently updated.^8^

Known postnatal risk factors for BPD development include low gestational age (GA), prolonged mechanical ventilation and oxygen exposure.^3^ However, BPD has also been described in preterm infants who had never received invasive mechanical ventilation.^9^ Antenatal factors, including placental dysfunction, intrauterine growth restriction, chorioamnionitis, preeclampsia, maternal hypertension and smoking have been described as factors associated with an increased risk of BPD.^10^ The pathophysiology of how these factors contribute to BPD is complex and yet to be determined. The aetiology of BPD is thought to be multifactorial, with both antenatal and postnatal factors playing a significant role in the abnormal alveolarisation and pulmonary vascular remodelling seen in histology samples of preterm infants who died with BPD.^11^

BPD rates vary significantly between different centres and countries.^12^–^16^ In a retrospective cohort study including all levels of neonatal intensive care units (NICUs) within the California Perinatal Quality Care Collaborative, the overall combined BPD and death rate hugely varied from 17.7–73.4%.^13^ In addition, the rate of BPD and death was the highest in level II NICUs compared with level III and level IV NICUs.^17^ The variations may be explained by altitude and different local practices.^14^^,^^15^

Approximately 10% of births in Oman occur before 37 weeks of gestation.^18^ To the best of the authors’ knowledge, there are no studies that have investigated the prevalence, patterns of severity and risk factors for BPD in preterm infants in Oman. Therefore, this study aimed to determine the rate of occurrence and severity patterns of BPD and, secondarily, to identify major antenatal and postnatal factors associated with BPD in preterm infants <32 weeks of gestation admitted to a single, level III NICU in Oman.

## Methods

This retrospective observational study was conducted in the level III, 24-bed capacity NICU of Sultan Qaboos University Hospital, Muscat, Oman, recording approximately 5,000 births per year.

Eligible participants included preterm infants <32 weeks of gestation, admitted into the NICU between January 2010 and December 2017. Infants were excluded if they were transferred to other health institutions before 36 weeks of PMA.

Pre-determined datasets were collected from the electronic charts of the patients, including antenatal factors, birth information, type of ventilation received, intubation and extubation variables, duration of invasive mechanical ventilation, post-extubation support, respiratory status at 28 days of life and PMA of 36 weeks, other neonatal comorbidities and discharge condition. Antenatal factors included maternal age and maternal morbidities such as pre-eclampsia, sepsis and chorioamnionitis. Birth information included mode of delivery, gender, gestational age, birth growth parameters, Apgar scores and resuscitation at birth. Other neonatal comorbidities included intraventricular haemorrhage (IVH), necrotising enterocolitis (NEC) and BPD. Discharge conditions included PMA at discharge, weight and head circumference, oxygen therapy and nutritional support.

The primary outcomes of this study were the rate, severity patterns of BPD and the combined mortality and BPD rate in preterm infants <32 weeks of gestation. Factors linked to BPD as well as factors that highly predicted BPD were the secondary outcomes of this study.

The definition and severity of BPD were based on the most recent update of the NICHD workshop definition, which was published in 2018.^8^ In this updated scheme, BPD is defined as oxygen and/or respiratory support at a PMA of 36 weeks. The severity is graded into grades 1, 2 and 3 based on the type of respiratory support and oxygen concentration. No BPD is defined as at a PMA of 36 weeks, the infant is already discharged home in room air or is still in hospital but already in room air. Grade I BPD is defined as an infant at a PMA of 36 weeks who is on nasal continuous positive airway pressure (NCPAP)/nasal intermittent positive pressure ventilation (NIPPV)/cannula ≥3 L/ min with an FiO_2_ of 21%, nasal cannula flow 1–2 L/ min with an FiO_2_ of 22–29% or nasal cannula <1 L/ min with an FiO_2_ of 22–70%. Grade II BPD is defined as an infant at a PMA of 36 weeks, the infant is on intermittent positive pressure ventilation (IPPV) with an FiO_2_ of 21%, NCPAP/NIPPV/nasal cannula ≥3 L/ min with an FiO_2_ of 22–29%, nasal cannula 1–2 L/min with an FiO_2_ ≥30% or nasal cannula <1 L/min with FiO_2_ >70%. Grade III BPD is defined as an infant at a PMA of 36 weeks, the infant is on IPPV with an FiO_2_ >21% or NCPAP/NIPPV/nasal cannula ≥3 L/min with an FiO_2_ ≥30%.

Statistical Package for Social Sciences (SPSS), Version 23 (IBM Corp., Armonk, New York, USA) was used for data analysis. The study population was divided into two groups: BPD and no BPD. Continuous variables were expressed as mean ± standard deviation or median and interquartile range. Categorical variables were expressed as frequency and percentages. Chi-squared and Fisher’s exact tests were used to assess differences between the categorised variables and small frequencies. Independent sample t-tests or Mann-Whitney U tests were used to test parametric and non-parametric data, respectively. To estimate the risk of BPD for different factors, odds ratios (OR) and 95% confidence intervals (CI) were obtained. A binary logistic regression analysis was performed to identify the significant predictors for BPD. Additional analysis of primary outcomes based on stratification classified the infants into two gestational-age categories, 22–28 weeks and 29–31 weeks. A *P* value of <0.05 was considered statistically significant.

Ethical approval for the study was obtained through the institutional medical research ethics committee (MREC#1887). Consent was waived due to the retrospective nature of the study.

## Results

**Figure 1:**
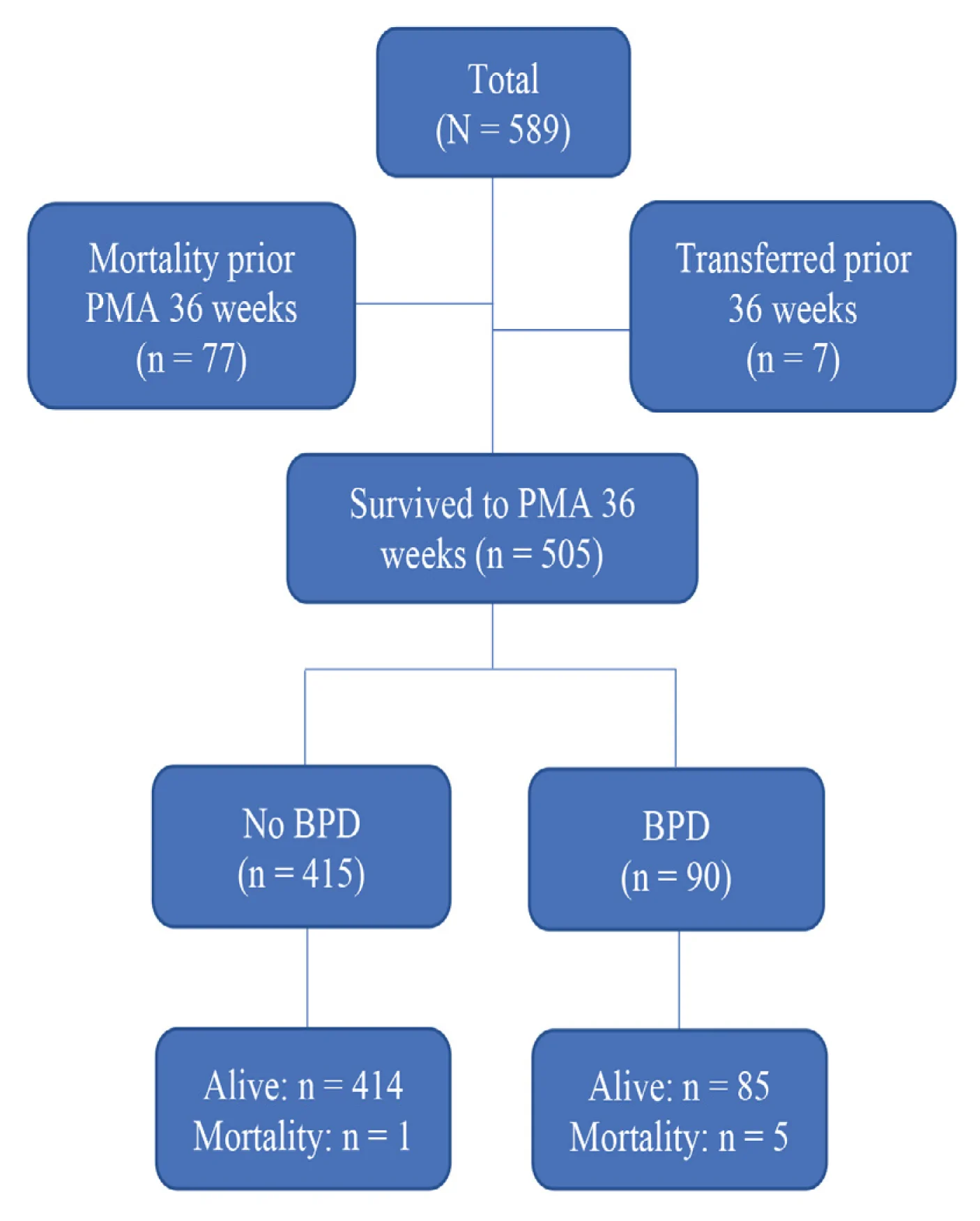
Flowchart showing participant selection. PMA = post-menstrual age; BPD = bronchopulmonary dysplasia.

**Figure 2:**
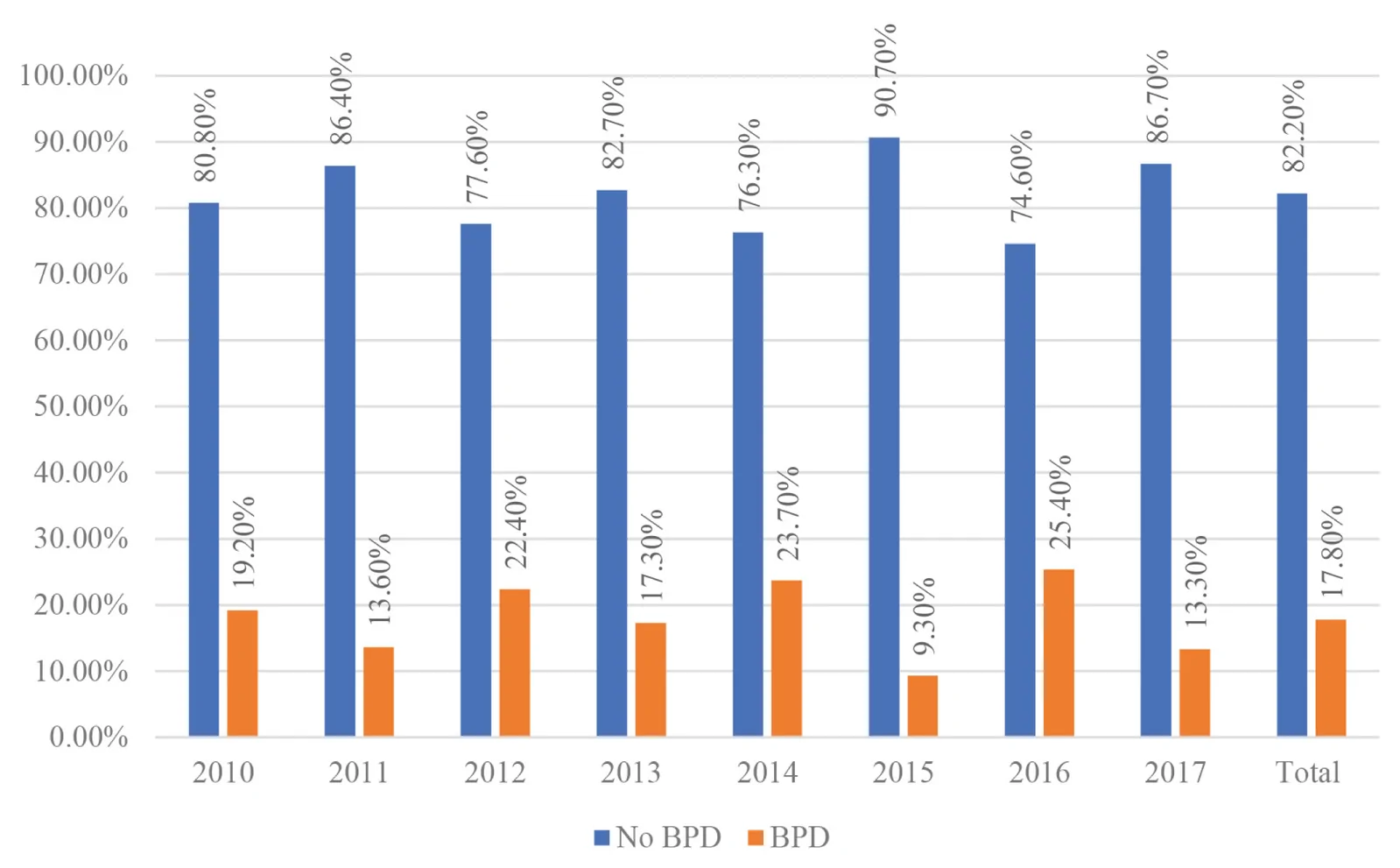
Yearly bronchopulmonary dysplasia rate in infants surviving to a post-menstrual age of 36 weeks during the study period. BPD = bronchopulmonary dysplasia.

A total of 589 preterm infants with gestational ages <32 weeks were admitted to the NICU during the study period [Figure 1]. Of these, 83 infants died (mortality rate = 14.1%). BPD was diagnosed in 90 out of 505 infants who survived up to a PMA of 36 weeks (17.8%). The combined mortality and BPD rate in this study was 28.4% [Table 1]. Grade 1, 2 and 3 BPD constituted 77.8% (n = 70), 7.8% (n = 7) and 14.4% (n = 13), respectively. When infants were stratified into two gestational age categories, 22–28 and 29–31 weeks, the BPD rate was 36.0% and 6.2%, respectively (*P* <0.0001). Similarly, the mortality rate was significantly higher in the 22–28 weeks category (24.7% versus 5.8%; *P* <0.0001). The combined BPD and/or mortality rate was also significantly higher in the 22–28 weeks GA category (51.0% versus 10.6% *P* <0.0001). However, no significant differences were found in the severity grades of BPD between the two GA categories [Table 2]. Further analysis of the rate of BPD in each year was performed. The yearly BPD rate ranged between 9.3–25.4% of infants who survived to a PMA of 36 weeks [Figure 2].

No significant differences were observed in maternal age, maternal complications, antenatal steroid use or method of birth. Significantly more infants in the BPD group needed intubation as part of the resuscitation at birth (83.3% versus 45.3%, *P* <0.001). Infants with BPD had significantly lower median gestational age (26 versus 29 weeks; *P* <0.001), birth weight (830 g versus 1,150 g; *P* <0.001), head circumference (23.5 cm versus 26 cm, *P* <0.001) and Apgar score at 1 minute and 5 minutes (5 versus 6; *P* <0.001) and (8 and 8; *P* <0.001), respectively. Invasive mechanical ventilation was received by 98.9% of the infants with BPD compared to only 58.3% of those without BPD (*P* <0.001). Infants with BPD were extubated at a median of 29 days of life, compared to a median of 2 days of life in those without BPD (*P* <0.001). Infants with BPD had a significantly longer median duration of mechanical ventilation (28 days versus 2 days; *P* <0.001). A total of 36 (40.0%) infants with BPD received ≥2 episodes of mechanical ventilation compared to 42 (17.5%) infants without BPD (*P* <0.001). At day 28 of life, all infants with BPD were still on respiratory support compared to only 30.6% of those without BPD (*P* <0.001) [Table 3].

The need for surfactant replacement therapy at 2 or more doses was higher in infants with BPD (95.6% versus 53.0%; *P* <0.001). Similarly, the rates of other prematurity complications, including pulmonary haemorrhage, IVH, patent *ductus arteriosus* (PDA), NEC and sepsis, were significantly higher in infants with BPD. The need for dexamethasone for extubation was similarly significantly higher in infants with BPD (33.3% versus 1.2%; *P* <0.001). The mortality rate after a PMA of 36 weeks was noted to be 5.6% in infants with BPD, which is significantly higher than that of infants without BPD (0.2%; *P* <0.001). Infants with BPD had a longer duration of NICU stay, as they were discharged at a median PMA of 40 weeks compared to 35 weeks in those without BPD (*P* <0.001). At discharge, infants with BPD had significantly higher weight and head circumference [Table 4]. Approximately 75% of infants without BPD were already discharged at a PMA of 36 weeks. This is compared to 100% of those with BPD who were still inpatients [Table 3].

Binary logistic regression analysis for the variables associated with BPD showed that factors that significantly predicted BPD were duration of mechanical ventilation (OR = 1.097, 95% CI: 1.097–1.173; *P* = 0.007), IVH grades 1 and 2 (OR = 4.19, 95% CI: 1.218–14.461; *P* = 0.023) and NEC stages II and III (OR = 5.272, 95% CI: 1.042–26.675; *P* = 0.044). Other factors that were associated with BPD, such as gestational age, birth weight, resuscitation at birth, Apgar scores, number of episodes of mechanical ventilation, surfactant use, pulmonary haemorrhage, PDA, sepsis and age at intubation and extubation, did not significantly predict BPD [Table 5].

## Discussion

To the best of the authors’ knowledge, this is the first study investigating BPD rate, severity and risk factors among at-risk preterm infants (<32 weeks’ GA) in Oman. The study found that the BPD rate, defined as oxygen and/or respiratory support at a PMA of 36 weeks, is 17.8% and the combined death and BPD rate is 28.4%. Most of the BPD cases were grade 1. This study did not identify any significant antenatal risk factors related to BPD. However, multiple perinatal and postnatal factors were associated with BPD, such as lower birth weight, lower GA at birth, lower Apgar scores, resuscitation at birth, mechanical ventilation and longer duration of mechanical ventilation. Other prematurity comorbidities were also associated with BPD.

The BPD rate in the current study’s NICU is comparable to other centres across the world, falling within the broad global incidence range of 17–75%, using the same definition of oxygen and/or respiratory support at 36 PMA.^19^ However, worldwide, the reported rates of BPD vary widely depending on the range of gestational age and birth weight of the population of preterm infants included in the studies.^20^–^22^ For example, in a single-centre US study by Sharma *et al*., which reported on BPD in 263 extremely preterm infants (23–27 weeks), 58.9% of infants were on oxygen and/or respiratory support at a PMA of 36 weeks.^23^ In comparison, in a single-centre Korean study that included 629 preterm infants younger than 30 weeks GA, admitted between 2009 and 2018, 13.8% of infants were on oxygen or respiratory support at 36 weeks PMA.^24^

The prevalence of BPD also varies across centres in multi-centre studies.^17^^,^^24^ In a multicentre US study that included 15,779 infants born between 22 and 29 weeks across 116 NICUs within the California Perinatal Quality Care Collaborative, approximately 33% of survivors with a PMA of 36 weeks were either still in the hospital on oxygen or discharged home on oxygen.^17^ NICU level of care may affect the prevalence of BPD, as level II NICUs seem to have higher rates of BPD compared to level III.^17^ This variation may be related to the level of experience and variation in practices in the management of preterm infants at risk of BPD.^25^^,^^26^

The current study did not identify any antenatal factors significantly related to BPD, as no significant differences were observed in maternal age and maternal morbidities such as pregnancy-induced hypertension, pre-eclampsia and chorioamnionitis. These findings correlate with Morrow *et al*., who showed similar findings except for maternal smoking status and preexisting hypertension, which significantly correlated with BPD.^27^ Maternal smoking was not investigated in this study considering that female smoking is generally rare in the Omani population.^28^ Likely risks for BPD are related to neonatal factors as demonstrated in the current study. This study identified various perinatal and postnatal factors associated with BPD, including lower GA, lower birth weight, need for resuscitation at birth, lower Apgar scores at 1 and 5 minutes, need for mechanical ventilation, longer duration of mechanical ventilation, need for respiratory support at 28 days, surfactant therapy, pulmonary haemorrhage, IVH, PDA, NEC and need for dexamethasone for extubation. Previous studies have also shown similar associations.^29^–^32^ This is possibly explained by the prematurity of the anatomy and physiology of these neonates.

In the current study, NEC stages II and III significantly predicted BPD. This is likely related to the fact that the two conditions shared a common physiological factor—inflammation. The relationship between NEC and BPD and the hypothesis of the gutlung axis has been extensively reviewed recently.^33^ The hypothesis postulates a crosstalk between the gastrointestinal tract and respiratory tract at various levels such as the microbiome, immunity and metabolites. Intestinal dysbiosis, coupled with a dysregulated immune system, can mediate lung inflammation and injury through inflammatory cytokines and cellular immune signalling pathways. Ultimately, the lung inflammation occurring concurrently with NEC contributes to the development of BPD.^33^

The association between IVH and BPD has also been described in the literature. For instance, in Jassem-Bobowicz *et al*.’s study, IVH was significantly associated with BPD in their univariate analysis.^34^ However, to the best of the authors’ knowledge, no study has reported that IVH was predictive of BPD. Interestingly, although both IVH grades 1 and 2 and grades 3 and 4 were associated with BPD in the univariate analysis of this study, only grades 1 and 2 predicted BPD according to the binary regression analysis. This could be related to the survival effect because infants with more severe IVH tend to have higher mortality.^35^ Further prospective studies are recommended to validate the value of IVH as a factor in predicting the risk of developing BPD.

The current study serves as a foundation for assessing the magnitude of BPD in preterm infants in Oman. To ensure optimal outcomes and efficient allocation of necessary resources and interventions, a comprehensive multi-central collaborative study on BPD in Oman is of paramount importance. Such studies aid in the understanding of the risk factors, pathogenesis and long-term implications of the condition and also provide valuable insights into tailored treatment strategies and preventative measures. By identifying high-risk groups and prognostic indicators, healthcare providers can prioritise resources for early detection, intervention and ongoing support, ultimately improving the quality of life for affected infants and their families. In addition, research on BPD facilitates the development of innovative therapies and advances in neonatal care, enhancing the overall healthcare landscape for preterm infants. The continuous effort to study and comprehend BPD underscores the significance of evidence-based approaches in guiding clinical decisions and optimising resource allocation for the best possible outcomes.

The strength of this study is its relatively large population size of <32 weeks GA infants and the use of the updated NICHD’s BPD definition and classification system to define BPD. However, this study was subject to a few limitations. This study is a single-centre, retrospective and observational study with an unbalanced number of infants in the non-BPD and BPD groups, limiting the generalisability of the findings to other cohorts.

## Conclusion

The rate of BPD in a level III NICU in Oman was 17.8%, mostly classified as grade 1. BPD is significantly associated with lower birth weight, lower gestational age, lower Apgar scores, the need for intubation and invasive mechanical ventilation, pulmonary haemorrhage, PDA, sepsis and receiving dexamethasone. However, only longer durations of mechanical ventilation, grades 1 and 2 IVH and stages II and III NEC are predictors for BPD in this study. Accordingly, strategies to avoid invasive mechanical ventilation and limiting its duration may help decrease BPD rates. A multi-centre study including various level III and II NICUs in the country will provide valuable data on the overall prevalence of BPD in Oman. It will help in enabling further insights into the predictive values of IVH and NEC and provide valuable data for the optimal allocation of necessary resources and interventions for the prevention and management of BPD in Oman.

## Figures and Tables

**Table 1:** Primary outcomes of infants included in this study

Outcome	n (%)
BPD*	90 (17.8)
No BPD*	415 (82.2)
Total mortality alone†	83 (14.1)
Combined BPD and mortality†	167 (28.4)

BPD = bronchopulmonary dysplasia.

* Percentage out of the total number of infants who were alive at PMA 36 weeks (n = 505).

† Percentage out of the total number of infants (N = 589).

**Table 2:** Primary outcomes based on stratification of gestational age

Outcome	n (%)		*P* value
	GA 22–28 weeks	GA 29–31 weeks	
**BPD**	71 (36.0)*	19 (6.2)†	<0.0001
**Total mortality**	64 (24.7)‡	19 (5.8)§	<0.0001
**Combined BPD and/or mortality**	132 (51.0)‡	35 (10.6)§	<0.0001
**BPD grade**	n = 71	n = 19	
1	54 (76.1)	16 (84.2)	0.362
2	7 (9.8)	0 (0.0)	
3	10 (14.1)	3 (15.8)	

GA = gestational age; BPD = bronchopulmonary dysplasia.

* Percentage out of the total number of neonates GA 22–28 weeks who survived to PMA 36 weeks (n =197).

† Percentage out of the total number of neonates GA 29–32 weeks who survived to PMA 36 weeks (n = 308).

‡ Percentage out of the total number of neonates GA 22–28 (n = 259).

§ Percentage out of the total number of neonates GA 29–32 weeks (n = 330).

**Table 3:** Difference in patients’ antenatal, perinatal and post-natal characteristics (N = 505)

Characteristic	n (%)		*P* value
	No BPD (n = 415)	BPD (n = 90)	
**Median maternal age in years (IQR)**	28 (24–33)	29 (25–33)	0.909
**Maternal complications**			0.602
No complication	169 (40.7)	43 (47.8)	
PIH/pre-eclampsia	75 (18.1)	14 (15.6)	
Sepsis/ chorioamnionitis	22 (5.3)	3 (3.3)	
Others	149 (35.9)	30 (33.3)	
**Antenatal steroids**	258 (62.2)	55 (61.1)	0.946
**Mode of delivery**			0.579
SVD	144 (34.7)	37 (41.1)	
Instrumental	4 (1.0)	1 (1.1)	
Elective LSCS	2 (0.5)	1 (1.1)	
Emergency LSCS	265 (63.9)	51 (56.7)	
**Resuscitation**			<0.001
Routine	89 (21.4)	1 (1.1)	
PPV	135 (32.5)	9 (10)	
Intubation	188 (45.3)	75 (83.3)	
CPR	3 (0.7)	5 (5.6)	
**Median gestational age in weeks (IQR)**	29 (28–31)	26 (25–28)	<0.001
**Gender**			0.589
Male	224 (54.0)	52 (57.8)	
Female	191 (46.0)	38 (47.2)	
**Median birth weighting (IQR)**	1,150 (1,040–1,480)	830 (697–977)	<0.001
**Median birth HC in cm (IQR)**	26 (25–28)	23.5 (22–25)	<0.001
**Median Apgar at 1 min (IQR)**	6 (5–8)	5 (3–7)	<0.001
**Median Apgar at 5 min (IQR)**	8 (8–9)	8 (6–9)	<0.001
**Required invasive ventilation**	242 (58.3)	89 (98.9)	<0.001
**Median DOL at intubation (IQR)**	1 (1–1)	1 (1–1)	0.556
**Median DOL at extubation (IQR)**	2 (1–7)	29 (10–47)	<0.001
**Median duration of MV in days (IQR)**	2 (1–7)	28 (12–44)	<0.001
**Episodes of MV**			<0.001
1	198 (47.7)	53 (58.9)	
≥2	42 (10.1)	36 (40)	
**Respiratory status at day 28**			<0.001
Discharged	19 (4.6)	0 (0.0)	
Respiratory support	127 (30.6)	90 (100.0)	
Room air	269 (64.8)	0 (0.0)	
**Respiratory status at 36 weeks**			<0.001
Discharged	310 (74.7)	0 (0.0)	
MV	0 (0.0)	10 (11.1)	
CPAP	0 (0.0)	22 (24.4)	
HFNC	0 (0.0)	10 (11.1)	
LFNC	0 (0.0)	48 (53.3)	
Room air	105 (25.3)	0 (0.0)	

BPD = bronchopulmonary dysplasia; IQR = interquartile range; PIH = pregnancy induced hypertension; SVD = spontaneous vaginal delivery; LSCS = lower segment caesarean section; PPV = positive pressure ventilation; CPR = cardiopulmonary resuscitation; HC = head circumference; DOL = day of life; MV = mechanical ventilation; CPAP = continuous positive airway pressure; HFNC = high flow nasal cannula; LFNC = low flow nasal cannula.

**Table 4:** Prematurity comorbidities and discharge variables (N = 505)

Variable	n (%)		*P* value
	No BPD (n = 415)	BPD (n = 90)	
**Received surfactant**	220 (53.0)	86 (95.6)	<0.001
**Pulmonary haemorrhage**	12 (2.9)	13 (14.4)	<0.001
**IVH**			<0.001
Grades 1 & 2	18 (4.3)	16 (17.8)	
Grades 3 & 4	11 (2.7)	14 (15.6)	
**PDA**	45 (10.8)	46 (51.1)	<0.001
**Sepsis**	80 (19.3)	54 (60.0)	<0.001
**NEC**			<0.001
I	22 (5.3)	12 (13.3)	
II, III	5 (1.2)	17 (18.9)	
**Dexamethasone**	5 (1.2)	30 (33.3)	<0.001
**Outcome**			<0.001
Alive	414 (99.8)	85 (94.4)	
Expired	1 (0.2)	5 (5.6)	
**Median PMA at discharge in weeks (IQR)**	35 (34–35)	40 (38–42)	<0.001
**Median discharge weight in g (IQR)**	1,825 (1,675–1,995)	2,565 (2,052–2,952)	<0.001
**Median discharge HC in cm (IQR)**	30 (29–31)	32 (31–34.5)	<0.001
**Readmission**	87 (21.0)	39 (43.3)	<0.001

BPD = bronchopulmonary dysplasia; IVH = intraventricular haemorrhage; PDA = patent ductus arteriosus; NEC = necrotising enterocolitis; PMA = post-menstrual age; IQR = interquartile range; HC = head circumference.

**Table 5:** Binary logistic regression analysis

Variable	B	*P* value	OR (95% CI)
**Resuscitation**			
Routine			
PPV	1.224	0.425	3.401 (0.168–68.788)
Intubation	1.163	0.452	3.200 (0.154–66.530)
CPR	2.403	0.195	11.059 (0.293–417.802)
**Gestational age in weeks**	0.214	0.138	1.239 (0.933–1.645)
**Birth weight in g**	−0.001	0.128	0.999 (0.997–1.000)
**Apgar at 1 minute**	−0.146	0.256	0.864 (0.672–1.111)
**Apgar at 5 minutes**	0.074	0.647	1.077 (0.784–1.480)
**Episodes of mechanical ventilation**			
1			
≥2	−0.362	0.433	0.697 (0.282–1.721)
**Surfactant given**	0.235	0.847	1.265 (0.117–13.716)
**Pulmonary haemorrhage**	−0.311	0.619	0.733 (0.215–2.498)
**IVH**			
No IVH			
Grades 1 & 2	1.434	0.023	4.197 (1.218–14.461)
Grades 3 & 4	0.361	0.593	1.435 (0.381–5.400)
**PDA**	0.234	0.587	1.263 (0.543–2.937)
**Blood culture sepsis**	0.315	0.444	1.370 (0.612–3.070)
**NEC**			
No NEC			
NEC I	0.363	0.569	1.438 (0.413–5.011)
NEC II, III	1.662	0.044	5.272 (1.042–26.675)
**Dexamethasone**	0.111	0.900	1.117 (0.199–6.282)
**DOL at intubation**	−0.039	0.706	0.962 (0.785–1.178)
**DOL at extubation**	0.021	0.340	1.021 (0.978–1.067)
**Mechanical ventilation duration in days**	0.093	0.007	1.097 (1.026–1.173)

OR = odds ratio; CI = confidence interval; PPV = positive pressure ventilation; CPR = cardiopulmonary resuscitation; IVH = intraventricular haemorrhage; PDA = patent ductus arteriosus; NEC = necrotising enterocolitis; DOL = day of life.

## References

[1] Northway WH, Rosan RC, Porter DY (1967). Pulmonary disease following respirator therapy of hyaline-membrane disease. Bronchopulmonary dysplasia. N Engl J Med.

[2] Jobe AH (2011). The new bronchopulmonary dysplasia. Curr Opin Pediatr.

[3] Brener Dik PH, Nino Gualdron YM, Galletti MF, Cribioli CM, Mariani GL (2017). Bronchopulmonary dysplasia: Incidence and risk factors. Arch Argent Pediatr.

[4] Jobe AH, Bancalari E (2001). Bronchopulmonary dysplasia. Am J Respir Crit Care Med.

[5] Ryan RM (2006). A new look at bronchopulmonary dysplasia classification. J Perinatol.

[6] Jensen EA, Wright CJ (2018). Bronchopulmonary dysplasia: The ongoing search for one definition to rule them all. J Pediatr.

[7] Poindexter BB, Feng R, Schmidt B, Aschner JL, Ballard RA, Hamvas A (2015). Comparisons and limitations of current definitions of bronchopulmonary dysplasia for the prematurity and respiratory outcomes program. Ann Am Thorac Soc.

[8] Higgins RD, Jobe AH, Koso-Thomas M, Bancalari E, Viscardi RM, Hartert TV (2018). Bronchopulmonary dysplasia: Executive summary of a workshop. J Pediatr.

[9] Vliegenthart RJS, Onland W, van Wassenaer-Leemhuis AG, De Jaegere APM, Aarnoudse-Moens CSH, van Kaam AH (2017). Restricted ventilation associated with reduced neurodevelopmental impairment in preterm infants. Neonatology.

[10] Taglauer E, Abman SH, Keller RL (2018). Recent advances in antenatal factors predisposing to bronchopulmonary dysplasia. Semin Perinatol.

[11] Alvira CM (2016). Aberrant pulmonary vascular growth and remodeling in bronchopulmonary dysplasia. Front Med (Lausanne).

[12] Su BH, Hsieh WS, Hsu CH, Chang JH, Lien R, Lin CH (2015). Neonatal outcomes of extremely preterm infants from Taiwan: Comparison with Canada, Japan, and the USA. Pediatr Neonatol.

[13] Al-Essa M, Maiyegun SO (2004). The rate and pattern of bronchopulmonary dysplasia in Kuwait. Ann Saudi Med.

[14] Jeon GW (2020). Changes in the incidence of bronchopulmonary dysplasia among preterm infants in a single center over 10 years. Neonatal Med.

[15] Rojas MX, Rojas MA, Lozano JM, Rondon MA, Charry LP (2012). Regional variation on rates of bronchopulmonary dysplasia and associated risk factors. ISRN Pediatr.

[16] Burgess-Shannon J, Briggs S, Oddie S, Mactier H (2023). Variation in use of extended pulse oximetry testing to guide decisions around home oxygen provision for ex-preterm infants; A nationwide survey of UK neonatal units. Respir Med Res.

[17] Lapcharoensap W, Gage SC, Kan P, Profit J, Shaw GM, Gould JB (2015). Hospital variation and risk factors for bronchopulmonary dysplasia in a population-based cohort. JAMA Pediatr.

[18] Sultanate of Oman Ministry of Health (2020). Birth & Death Facts Report.

[19] Siffel C, Kistler KD, Lewis JFM, Sarda SP (2021). Global incidence of bronchopulmonary dysplasia among extremely preterm infants: A systematic literature review. J Matern Fetal Neonatal Med.

[20] Trembath A, Laughon MM (2012). Predictors of bronchopulmonary dysplasia. Clin Perinatol.

[21] Valenzuela-Stutman D, Marshall G, Tapia JL, Mariani G, Bancalari A, Gonzalez A (2019). Bronchopulmonary dysplasia: Risk prediction models for very-low-birth-weight infants. J Perinatol.

[22] Tapia JL, Agost D, Alegria A, Standen J, Escobar M, Grandi C (2006). Bronchopulmonary dysplasia: Incidence, risk factors and resource utilization in a population of South American very low birth weight infants. J Pediatr (Rio J).

[23] Sharma A, Xin Y, Chen X, Sood BG (2020). Early prediction of moderate to severe bronchopulmonary dysplasia in extremely premature infants. Pediatr Neonatol.

[24] Choi CW, Kim BI, Kim EK, Song ES, Lee JJ (2012). Incidence of bronchopulmonary dysplasia in Korea. J Korean Med Sci.

[25] Gortner L, Misselwitz B, Milligan D, Zeitlin J, Kollee L, Boerch K (2011). Rates of bronchopulmonary dysplasia in very preterm neonates in Europe: Results from the MOSAIC cohort. Neonatology.

[26] Kardum D, Filipović-Grčić B, Müller A, Dessardo S (2019). Incidence and risk factors for moderate and severe bronchopulmonary dysplasia in very low birth weight infants in two Croatian perinatal regions – a retrospective cohort study. J Pediatr Neonatal Individualized Med.

[27] Morrow LA, Wagner BD, Ingram DA, Poindexter BB, Schibler K, Cotten CM (2017). Antenatal determinants of bronchopulmonary dysplasia and late respiratory disease in preterm infants. Am J Respir Crit Care Med.

[28] Al Riyami AA, Afifi M (2004). Smoking in Oman: Prevalence and characteristics of smokers. East Mediterr Health J.

[29] Hernandez-Ronquillo L, Tellez-Zenteno JF, Weder-Cisneros N, Salinas-Ramirez V, Zapata-Pallagi JA, da Silva O (2004). Risk factors for the development of bronchopulmonary dysplasia: A casecontrol study. Arch Med Res.

[30] Ding L, Wang H, Geng H, Cui N, Huang F, Zhu X (2020). Prediction of bronchopulmonary dysplasia in preterm infants using postnatal risk factors. Front Pediatr.

[31] Kim S-H, Han YS, Chun J, Lee MH, Sung T-J (2020). Risk factors that affect the degree of bronchopulmonary dysplasia: Comparison by severity in the same gestational age. PLoS One.

[32] Jung YH, Jang J, Kim H-S, Shin SH, Choi CW, Kim E-K (2019). Respiratory severity score as a predictive factor for severe bronchopulmonary dysplasia or death in extremely preterm infants. BMC Pediatrics.

[33] Willis KA, Ambalavanan N (2021). Necrotizing enterocolitis and the gut-lung axis. Semin Perinatol.

[34] Jassem-Bobowicz JM, Klasa-Mazurkiewicz D, Zawrocki A, Stefanska K, Domzalska-Popadiuk I, Kwiatkowski S (2021). Prediction model for bronchopulmonary dysplasia in preterm newborns. Children (Basel).

[35] Piccolo B, Marchignoli M, Pisani F (2022). Intraventricular hemorrhage in preterm newborn. Predictors of mortality. Acta Biomed.

